# Type I Interferon in Human Autoimmunity

**DOI:** 10.3389/fimmu.2014.00306

**Published:** 2014-06-30

**Authors:** Timothy B. Niewold

**Affiliations:** ^1^Division of Rheumatology and Department of Immunology, Mayo Clinic, Rochester, MN, USA

**Keywords:** interferons, systemic lupus erythematosus, Sjogren’s syndrome, multiple sclerosis, scleroderma, systemic, type I diabetes, autoimmune thyroid disease

The type I interferon system plays a critical role in host defense in health, and a growing body of literature suggests that type I interferon is a critical mediator of human autoimmune disease (1). Type I interferons function as a bridge between the innate and adaptive immune systems, and as such play an important role in setting thresholds for response against self antigens. Many investigators have focused on the role type I interferons play in autoimmune disease. This fascinating and rapidly growing body of literature encompasses many different autoimmune diseases, including systemic lupus erythematosus, type I diabetes, multiple sclerosis, and others. Type I interferons play differing roles in human autoimmune conditions. For example, in the autoimmune diseases, systemic lupus erythematosus and Sjogren’s syndrome, increased interferon alpha signaling plays a pathogenic role (2, 3). Interestingly, interferon beta is used as a therapeutic in multiple sclerosis, an autoimmune disease of the central nervous system (4). Both interferon alpha and beta signal through the same type I interferon receptor and share many similarities in downstream signaling, suggesting that the disparate activities of type I interferons in lupus and multiple sclerosis relate to differences in the underlying disease processes and immunoregulation in these two diseases. In this Research Topic, a series of articles provides a comprehensive overview of the various roles type I interferons play in autoimmune diseases, with a focus on human immunology.

This Research Topic features a number of Original Research Articles, including a study by Mavragani et al. examining type I interferon levels in the organ-specific autoimmune disorders type I diabetes and autoimmune thyroid disease (5). They demonstrate high type I interferon levels in both of these autoimmune conditions, supporting the idea that high levels of type I interferon are detectable in organ-specific autoimmune conditions in addition to systemic autoimmune disorders. Clark et al. investigate genetic polymorphisms in the interferon regulatory factor 5 (IRF5) gene (6). This gene has been associated with susceptibility to systemic lupus erythematosus (7), and they demonstrate four distinct promoter regions have differential activity. Ko et al. study type I interferon-induced gene expression in patients with systemic lupus erythematosus (8). They demonstrate that the expression of type I interferon-induced genes in lupus immune cells differs significantly between ancestral backgrounds, which corresponds to clinical differences in the disease between ancestral backgrounds. A Methods article by Feng et al. examines public domain gene expression data to document patterns of type I interferon-induced gene expression and infer both positive and negative regulation by transcription factors (9).

The Research Topic also features a number of Review Articles focusing on various disease states. Liu et al. review murine models of systemic lupus erythematosus that are interferon-inducible, providing model systems of autoimmunity related to type I interferon (10). Wu et al. review the role of type I interferon in systemic sclerosis, a distinct autoimmune disease characterized by thickening and fibrosis of the skin, which shares a type I interferon signature with other autoimmune conditions (11). Li et al. review the evidence supporting a role for type I interferon in the pathogenesis of Sjogren’s syndrome, spanning genetic associations, gene expression studies, and clinical features of the disease (12). Reder et al. review the contrasting role of type I interferon in multiple sclerosis and systemic lupus erythematosus and other autoimmune conditions (13). In multiple sclerosis, type I interferon levels are low (14), and administration of recombinant type I interferon is an effective treatment. They review the evidence supporting multiple sclerosis as a low interferon autoimmune disease, and speculate on immunological features that might underlie this striking difference. Shrivastav et al. review the role of nucleic acid receptors in type I interferon generation in systemic lupus erythematosus (15), a disease characterized by pathological activation of the type I interferon pathway. These articles taken together provide an overview of many of the ways type I interferons have been implicated in human autoimmune disease, providing a fascinating window into the biology of the human immune system gone wrong.

## Conflict of Interest Statement

The author declares that the research was conducted in the absence of any commercial or financial relationships that could be construed as a potential conflict of interest.
